# Supplementary figures and images for: The impact of chronic intermittent hypoxia on enzymatic activity in memory-associated brain regions of male and female rats
Source: Biol Sex Differ. 2025 Jan 31;16:5. doi: 10.1186/s13293-025-00688-6 (PMC11786371; doi:10.1186/s13293-025-00688-6)

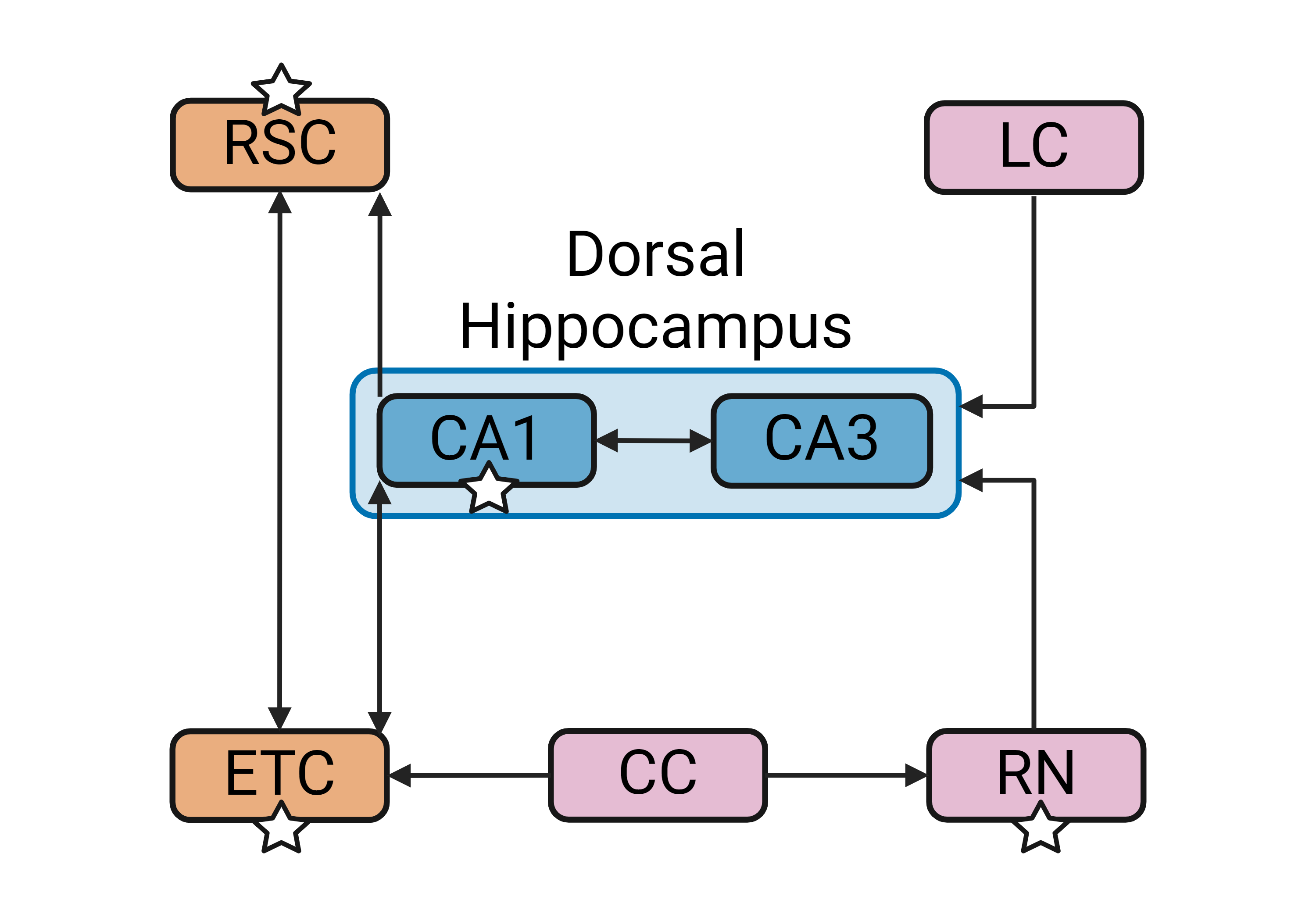

Supplement: Supplementary file 4 — Supplementary Material 4 [file 13293_2025_688_MOESM4_ESM.png]

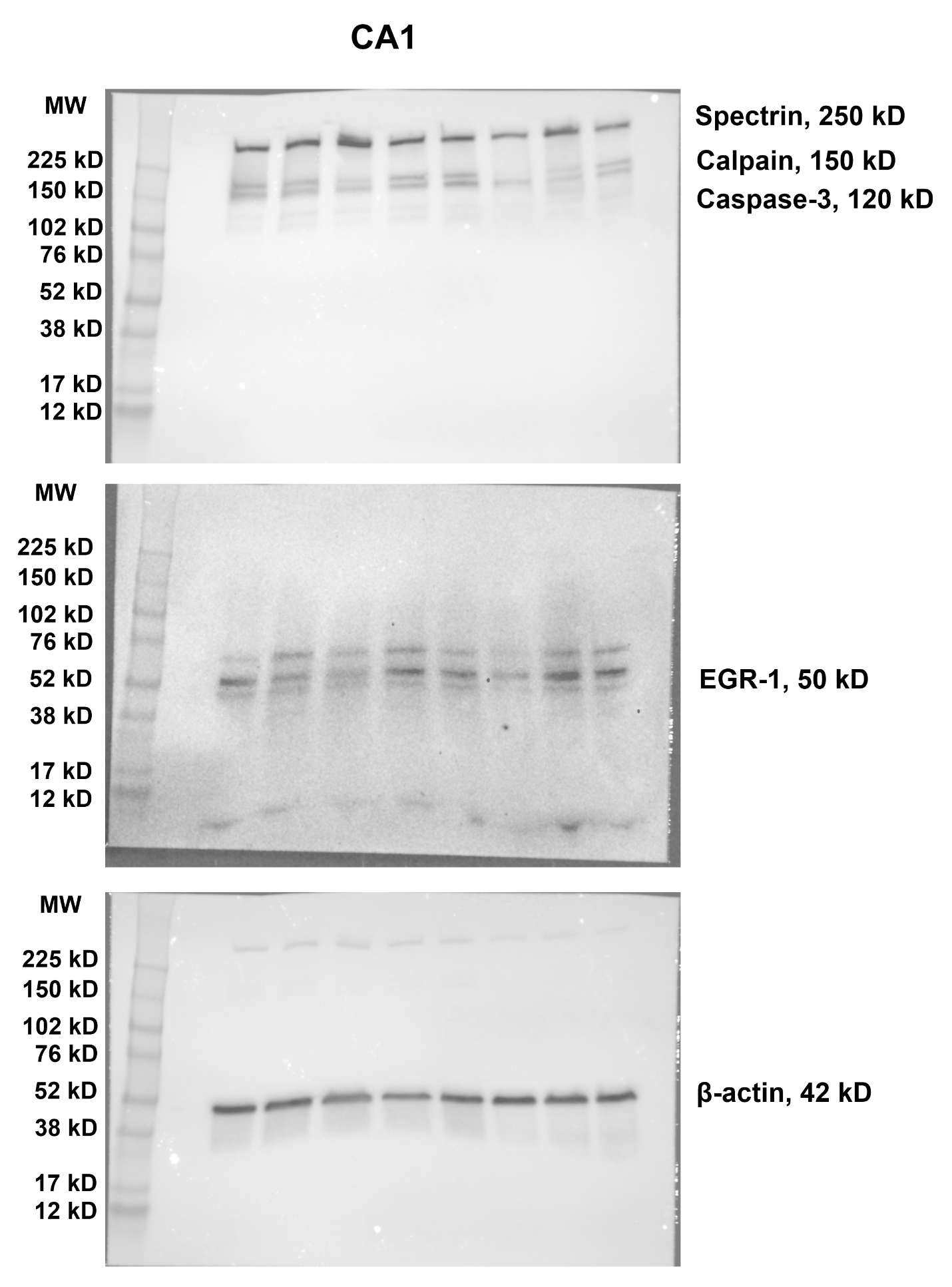

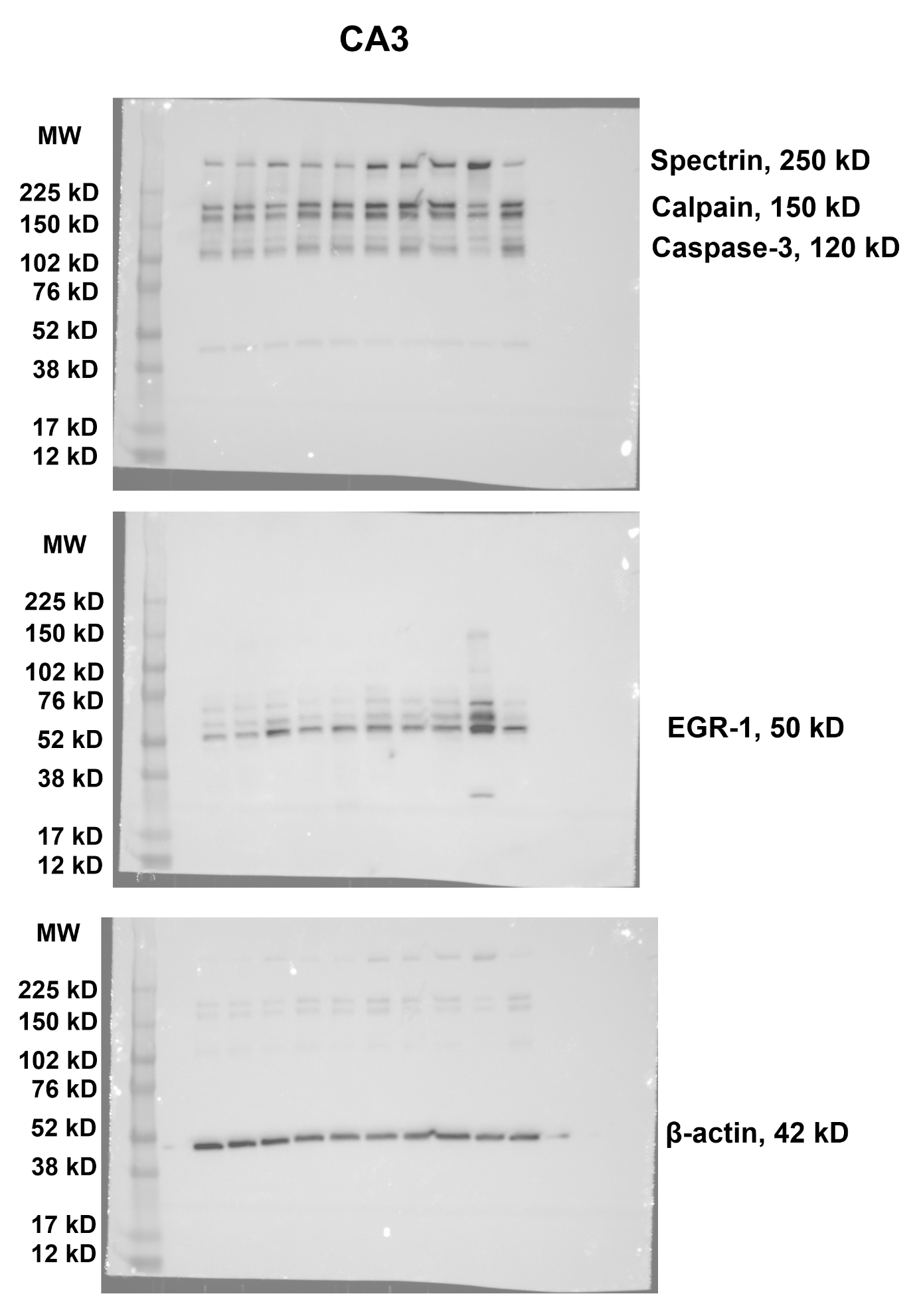

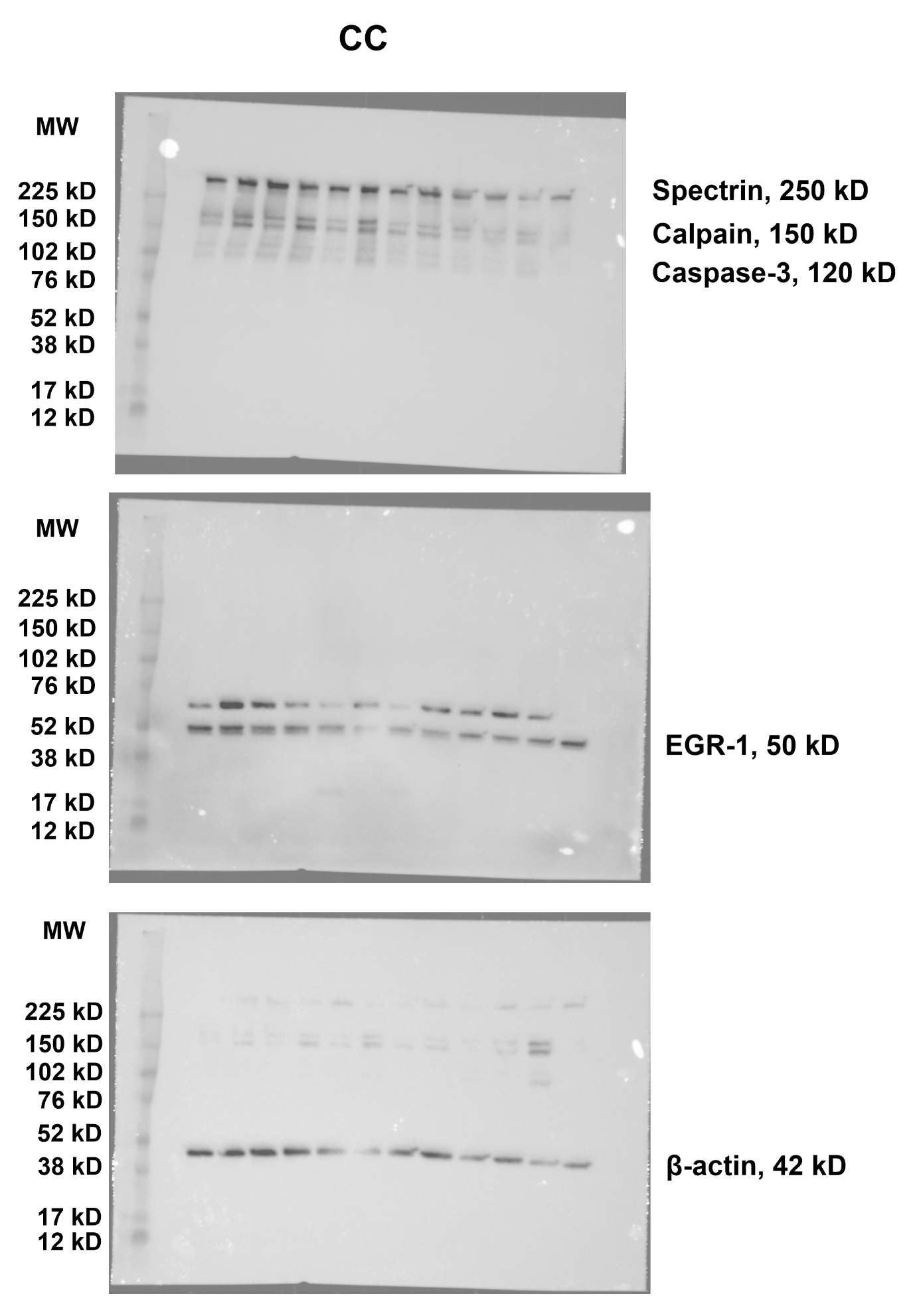

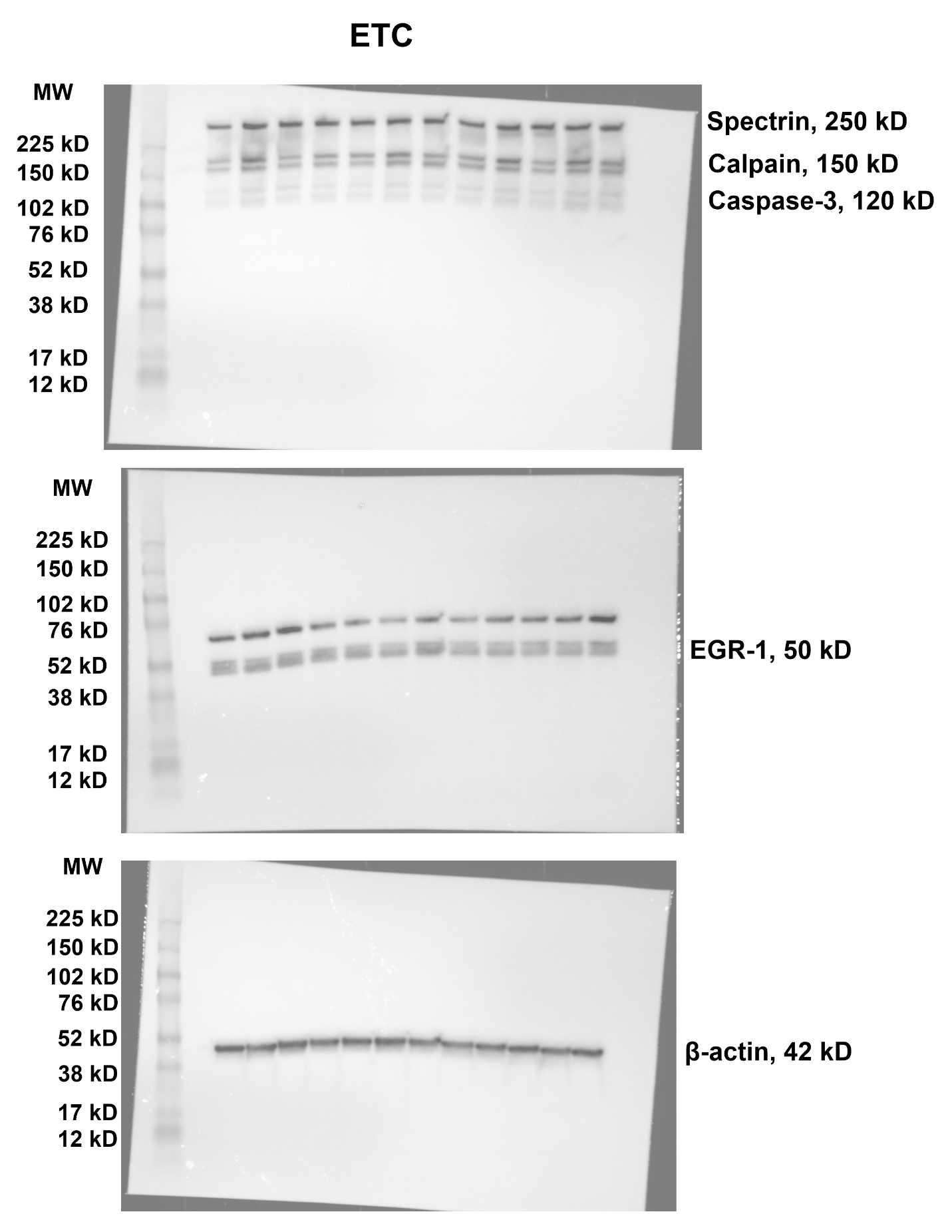

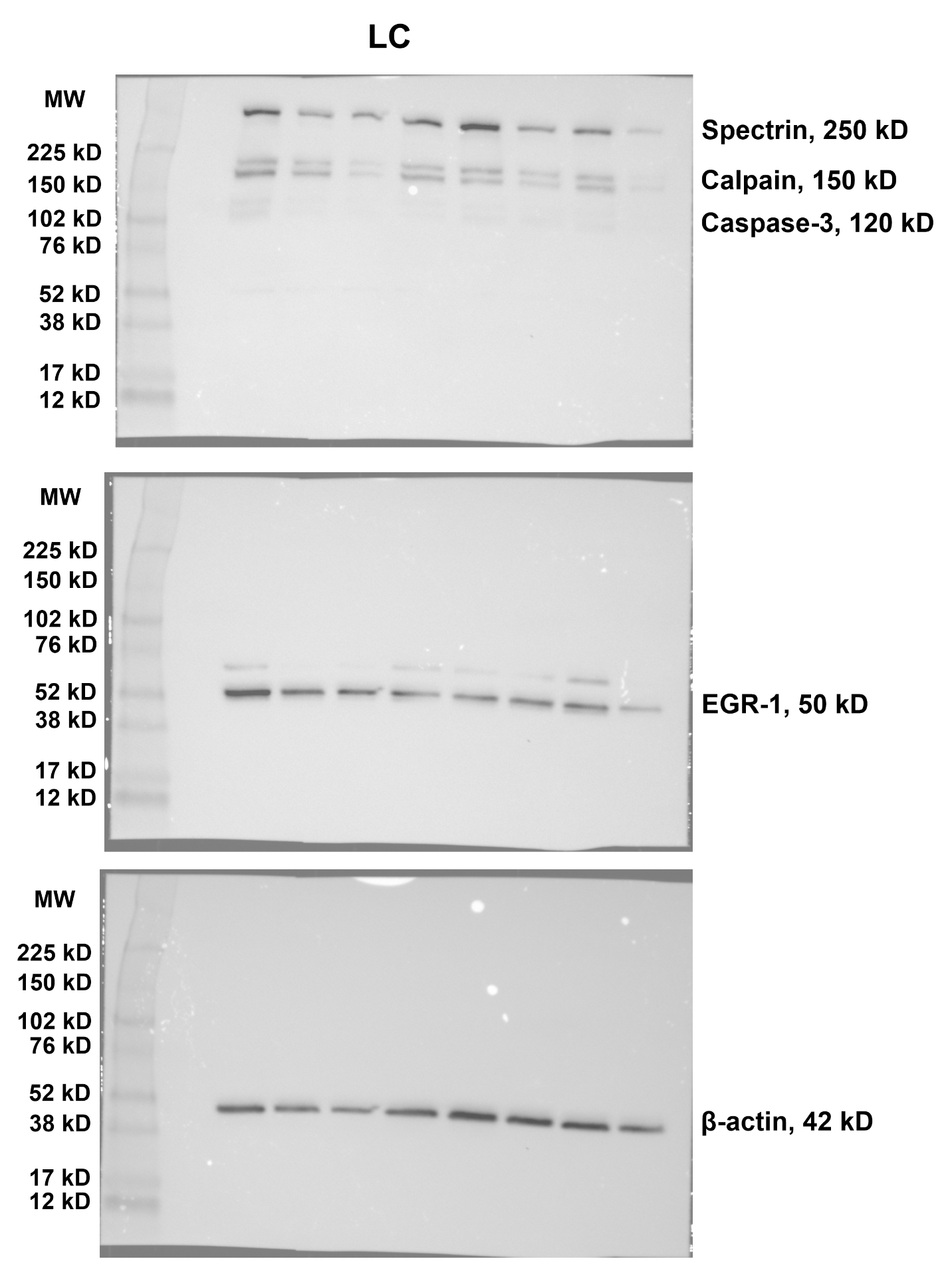

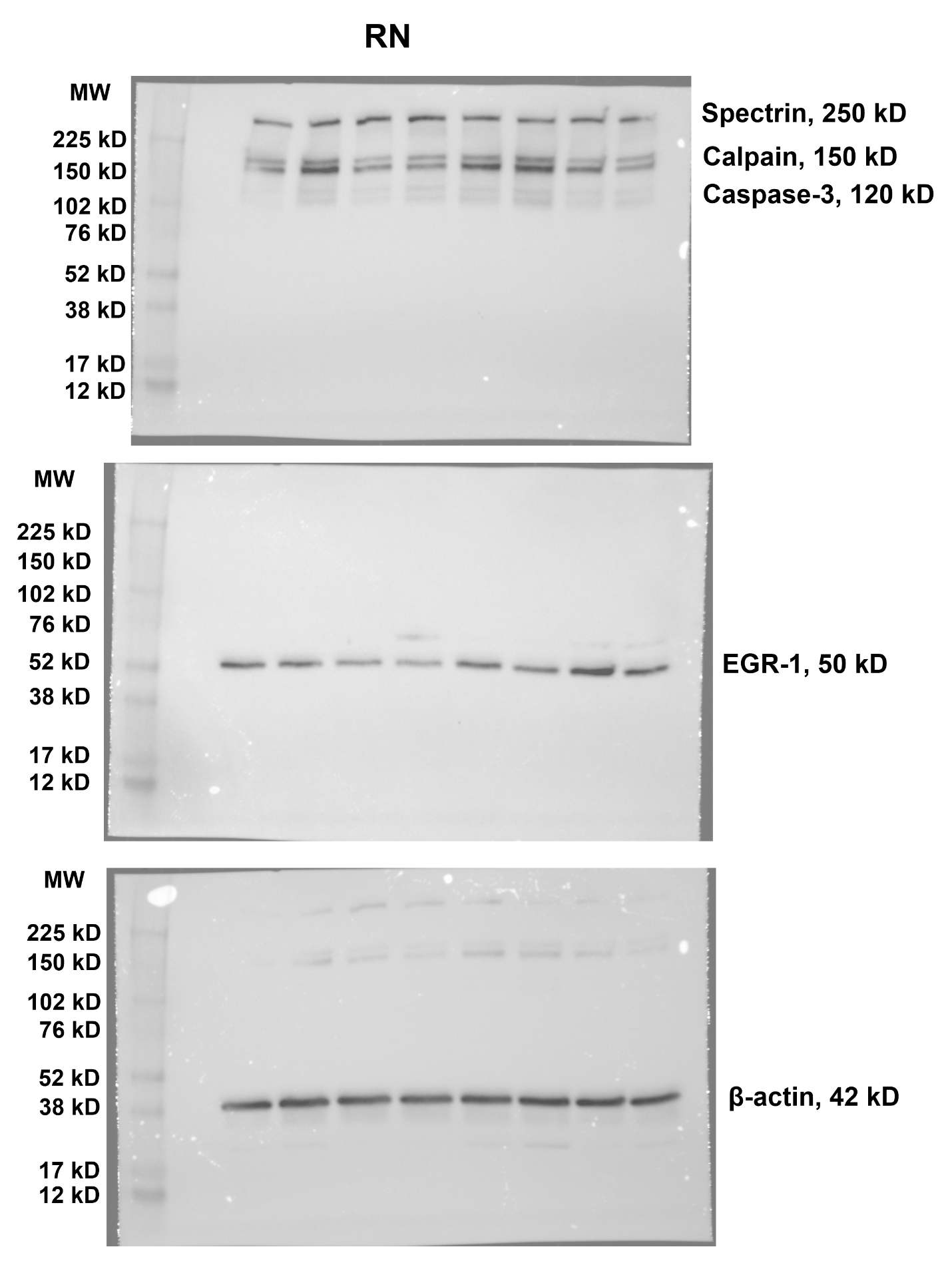

Supplement: Supplementary file 5 — Supplementary Material 5 [file 13293_2025_688_MOESM5_ESM.docx]
